# F/HN-pseudotyped lentiviral vector efficiently transduces non-human primate airways with no evidence of relevant toxicity

**DOI:** 10.1016/j.omta.2025.201655

**Published:** 2025-12-26

**Authors:** Uta Griesenbach, Gerry McLachlan, Anthony Sinadinos, Cédric Cheminay, Joseph Ashour, Jason Cox, Daniela Schwotzer, Kristin Vyhnal, Aradhana Gupta, Mario Chan, Cuixiang Meng, Kyriel Pineault, Emily Castells, Rebecca J. Dean, Mariana A. Viegas, A Christopher Boyd, Jane C. Davies, Deborah R. Gill, Stephen C. Hyde, Diann Blanset, Eric WFW. Alton

**Affiliations:** 1 National Heart and Lung Institute, Imperial College London, London SW3 6LR, UK; 2 UK Respiratory Gene Therapy Consortium, London SW3 6LR, UK; 3 The Roslin Institute, University of Edinburgh, Midlothian EH25 9RG, UK; 4 Boehringer Ingelheim Pharmaceuticals, Inc., Ridgefield, CT 06877, USA; 5 Lovelace Biomedical, Albuquerque, NM 87108, USA; 6 Radcliffe Department of Medicine, University of Oxford, Oxford OX3 9DS, UK; 7 Centre for Genomic and Experimental Medicine, Institute of Genetics & Cancer, University of Edinburgh, Edinburgh EH4 2XU, UK

**Keywords:** cystic fibrosis, gene therapy, lentiviral vector, lung gene transfer, non-human primates, transduction efficiency, toxicity

## Abstract

We have developed a third-generation lentiviral vector pseudotyped with Sendai virus F and HN envelope proteins (rSIV.F/HN) expressing functional cystic fibrosis transmembrane conductance regulator (CFTR) as a gene therapy for cystic fibrosis (BI 3720931). Here, we assessed transduction efficiency and acute toxicology of the rSIV.F/HN vector expressing an enhanced green fluorescent protein (EGFP) reporter gene in non-human primates (NHPs). Intubated male cynomolgus monkeys received one aerosolized dose of vector (*n* = 3) or placebo (*n* = 3). Toxicology was assessed by histopathology, clinical pathology, cytokine levels, and changes in body and organ weight. Transduction efficiency was quantified by EGFP immunohistochemistry in airway epithelial cells and vector-specific mRNA and DNA in the lung 7 days post-dosing. There were no vector-related clinical observations, mortalities, or changes in body or organ weight. Clinical pathology and cytokine analyses were unremarkable. Minimal mixed-cell centriacinar inflammation was observed in 1/3 vector-treated animals. Airway epithelial cell transduction efficiency was 9%–12%. Genomic DNA vector integration was detected in 6.7% of lung epithelial cells. Vector-specific mRNA levels were ∼45× endogenous CFTR mRNA levels in lung epithelium and ∼16× in bronchial brushings. This study extends earlier findings of rSIV.F/HN-based *in vivo* gene transfer in mice to NHPs, demonstrating transduction efficiency without relevant toxicity.

## Graphical abstract

**Figure:**
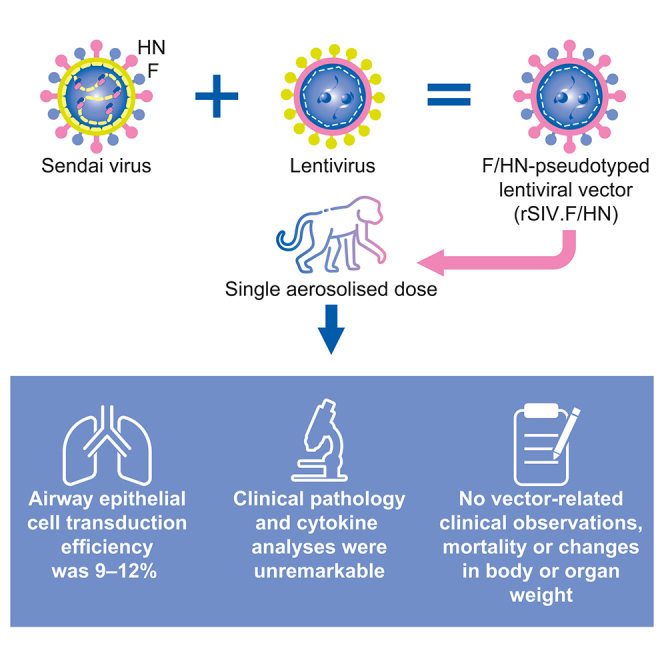

## Teaser

The lentiviral vector rSIV.F/HN has been developed for use as a gene therapy for cystic fibrosis (BI 3720931). Griesenbach and colleagues show that it efficiently transduces the respiratory airway epithelium of non-human primates, with no evidence of relevant toxicity. These findings support the progression of BI 3720931 toward the clinic.

## Introduction

Despite the unprecedented impact that cystic fibrosis transmembrane conductance regulator (CFTR) modulator therapies have had on cystic fibrosis (CF) clinical care, an unmet therapeutic need remains for a significant proportion of patients (10%–15% of the global CF population) who are genetically ineligible for, or intolerant of, CFTR modulator therapies.^1^ Gene therapy is a mutation-agnostic approach that may benefit patients from this population.^2^ However, limitations in pulmonary gene transfer have hindered the development of a gene therapy for CF that is suitable for clinical use.^3^ In a phase 2b non-viral gene therapy trial, repeated delivery of the CFTR complementary DNA (cDNA) complexed to the cationic lipid GL67A was shown to be safe and produced statistically significant improvement in lung function compared with placebo.^4^ At that time, however, the magnitude of efficacy was insufficient to warrant further development, highlighting the need for more potent vectors.^5^ Viral vectors offer the advantage of improved gene delivery.

Although adenoviral vectors have natural tropism for the lung, they are not suitable for CF gene therapy due to their short duration of expression and high immunogenicity, which prevents successful re-administration.^6^^,^^7^ Similarly, acquired immune responses limit the efficacy of adeno-associated viral (AAV) vectors after repeated administration.^8^ Additionally, AAV vectors have a limited packaging capacity^8^ and cannot carry the full-length *CFTR* coding sequence plus the required promoter/enhancer elements, necessitating the use of a truncated *CFTR* gene. AAV vectors carrying such transgenes have been assessed in CF models (mice and patient-derived organoids)^9^; however, the impact of these truncations on CFTR function in humans is unclear. A phase 1/2 trial of 4D-710, an AAV vector of this kind, in adults with CF is currently ongoing (NCT05248230).^10^ Another AAV vector-based gene therapy, SP-101, is also undergoing development in the phase 1/2 SAAVe trial in adults with CF (NCT06526923).^11^ Other viral vectors for CF gene therapy are also in development, such as the replication-defective herpes simplex virus (HSV)-based HSV-1 vector KB407, which is currently being assessed in a phase 1 clinical trial in adults with CF (NCT05504837).^12^

Sendai virus-mediated gene transfer to airway epithelial cells via the apical membrane is highly efficient.^13^^,^^14^ However, due to their short duration of expression and strong immunogenicity,^15^ Sendai virus vectors, similar to adenoviral and AAV vectors, have not been useful for CF gene therapy to date. Lentiviral vectors are capable of maintaining longer duration of expression through stable integration into the host genome.^16^ Although lentiviruses have evolved to transduce T cells via the gp120 protein on the envelope surface,^17^ most lentiviral vectors have been pseudotyped with vesicular stomatitis virus (VSV)-G glycoproteins to expand vector tropism.^18^ In the airways, receptors for VSV-G are lacking at the apical membrane of airway epithelial cells; thus, gene transfer efficiency is generally poor following topical administration,^16^ unless airway conditioning approaches are used prior to vector delivery.^19^^,^^20^

To address this, we have pseudotyped a lentiviral vector (using a simian immunodeficiency virus [SIV]) with Sendai virus F and HN envelope proteins (rSIV.F/HN) to allow for more efficient vector entry and gene transfer to airway epithelial cells, with a long duration of expression and without the need for pre-airway pre-conditioning.^3^ The addition of the HN envelope protein has also been shown to enhance transduction efficiency of VSV-G-pseudotyped vectors *in vitro*.^21^ The rSIV.F/HN vector, carrying a reporter gene to allow confirmation of transduction efficiency, has previously demonstrated efficient and persistent (∼2 years after a single dose) gene expression in rodent airways, with the ability to retain efficacy when readministered.^22^ Here, we present the results of a study assessing the safety and efficacy of the rSIV.F/HN vector in non-human primates (NHPs).

## Results

### Aerosol development results

The mean delivery efficiency using Tris, sodium, sucrose, and mannitol (TSSM) was 25% (standard deviation: 2.6%). Similarly, the mean delivery efficiency for the viral formation was 23% (standard deviation: 2.6%). The average transduction efficiency of the aerosol condensates (calculated as post-delivery amount/pre-delivery stock) was 89.82% ± 14.39.

### Inhalation exposures and delivery/deposition amounts

The exposure duration ranged from 5.5 to 5.8 min for control animals treated with diluent and from 5.3 to 6.3 min for vector-treated animals (Table 1). The delivered and deposited amount of vector formulation per animal was 1.0E9 transduction units (TU) and 2.5E8 TU, respectively.

**Table 1:** Exposure duration in control animals treated with TSSM diluent and vector-treated animals

Group	Animal	Exposure duration (min)
Diluent	Animal 1	5.75
	Animal 2	5.50
	Animal 3	5.50
SIV	Animal 1	5.50
	Animal 2	5.25
	Animal 3	6.25

Diluent refers to control animals treated with TSSM diluent (*n* = 3), while SIV refers to vector-treated animals (*n* = 3). SIV, simian immunodeficiency virus; TSSM, tris, sodium, sucrose, mannitol.

### Safety

**Figure 1:**
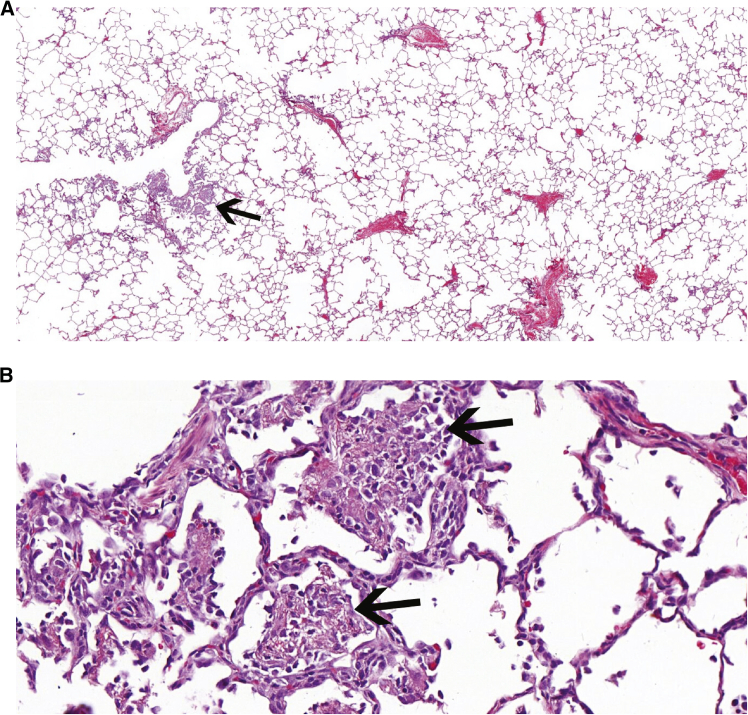
Histopathological observations following vector administration Minimal mixed-cell centriacinar inflammation observed in one of the three vector-treated animals. (A) Low-magnification image of the left cranial lung lobe (peripheral) showing focal area of change at the centriacinar region shown by arrow (hematoxylin and eosin stain [1.25x]). (B) High-magnification image of the area in Figure 2A showing area of mixed-cell inflammation shown by arrows (hematoxylin and eosin stain [10x]).

There were no vector-related clinical observations, mortality, or significant changes in body or organ weights. No clinical pathology findings were judged as biologically significant, and cytokine analyses showed no significant changes between vector- and diluent-treated animals. Minimal mixed-cell centriacinar inflammation was seen in one of the three vector-treated animals (Figure 1).

### Efficacy

**Figure 2:**
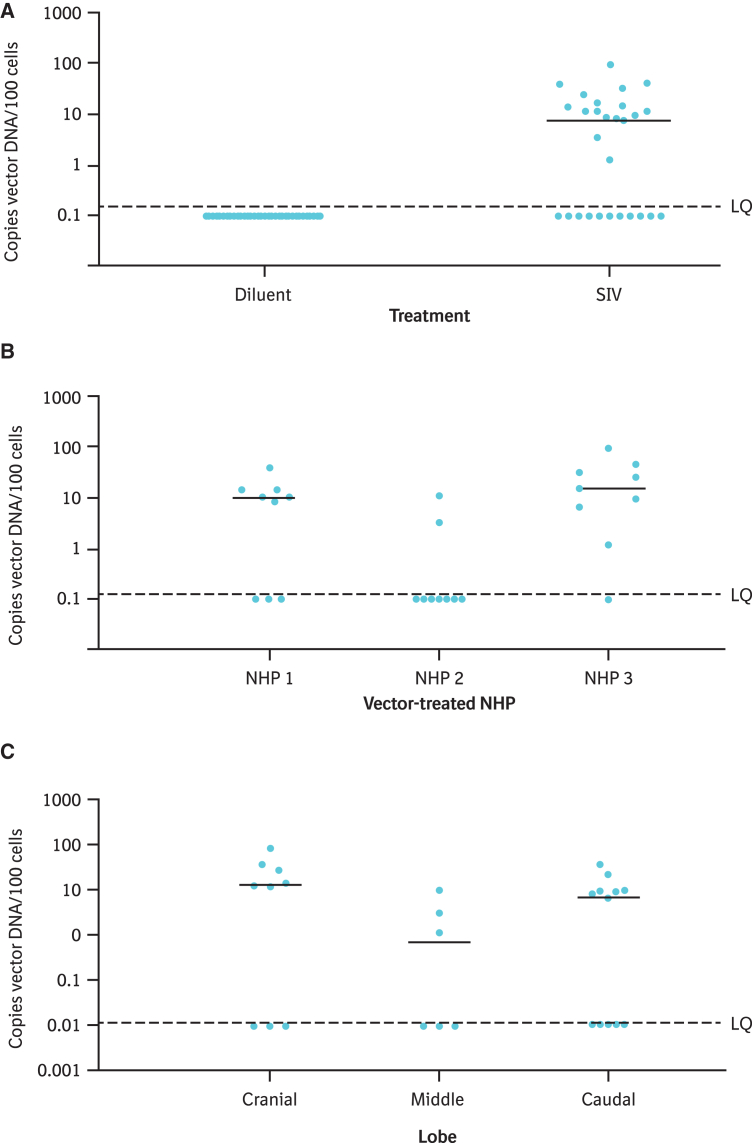
Vector integration into genomic DNA of lung epithelial cells Copies of vector DNA per 100 lung epithelial cells in (A) control animals treated with TSSM diluent (Diluent; *n* = 3) and vector-treated animals (SIV; *n* = 3) (nine samples were analyzed for each animal); (B) individual vector-treated animals (nine samples were analyzed for each animal); and (C) cranial (nine samples analyzed [three per animal]), middle (six samples analyzed [two per animal]) and caudal (12 samples analyzed [four per animal]) regions of the lung. LQ, limit of quantification; NHP, non-human primate; SIV, simian immunodeficiency virus; TSSM, tris, sodium, sucrose, mannitol.

**Figure 3:**
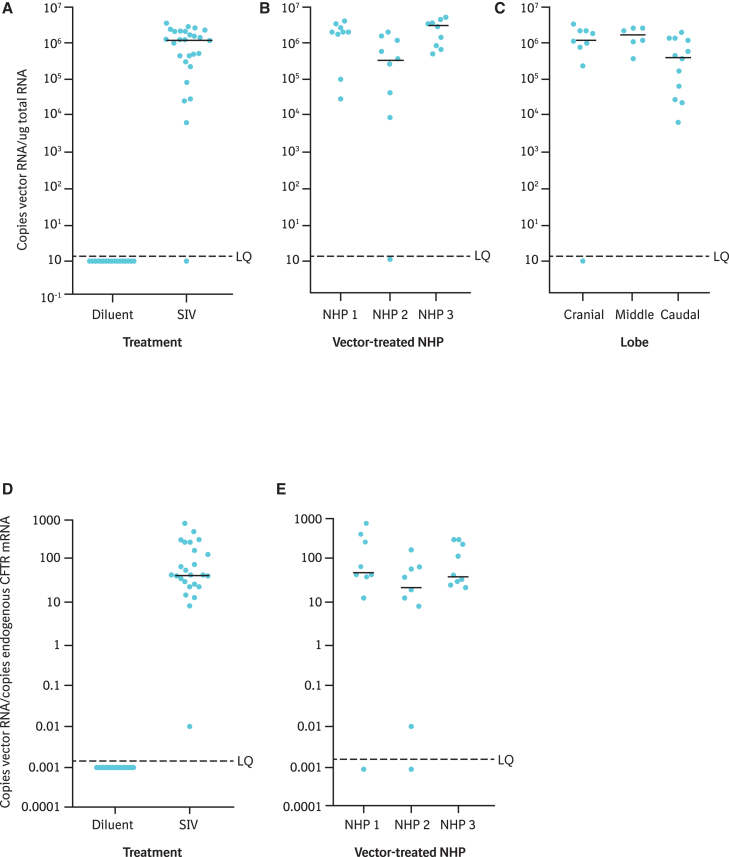
Vector-specific mRNA found in lung epithelial cells Copies of vector RNA per microgram of total RNA in lung epithelial cells from (A) control animals treated with TSSM diluent (Diluent; *n* = 3) and vector-treated animals (SIV; ***n*****= 3)** (nine samples were analyzed for each animal); (B) individual vector-treated animals; (C) cranial, middle, and caudal regions of the lung. Copies of vector RNA per copies of endogenous CFTR RNA in lung epithelial cells from (D) control animals treated with diluent (Diluent; *n* = 3) and vector-treated animals (SIV; *n* = 3) (nine samples were analyzed for each animal); (E) individual vector-treated animals. CFTR, cystic fibrosis transmembrane conductance regulator; LQ, limit of quantification; NHP, non-human primate; SIV, simian immunodeficiency virus; TSSM, tris, sodium, sucrose, mannitol.

**Figure 4:**
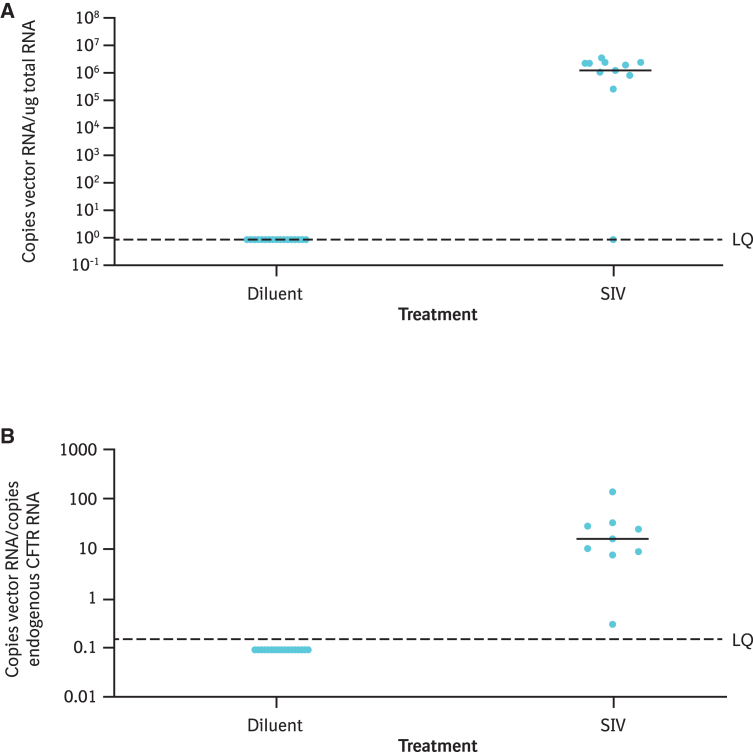
Vector-specific mRNA in bronchial brushings (A) Copies of vector RNA per microgram of total RNA and (B) copies of vector RNA per copies of endogenous CFTR RNA in bronchial brushings from control animals treated with TSSM diluent (Diluent; *n* = 3) and vector-treated animals (SIV; *n* = 3). Three bronchial brushings were taken from each animal. CFTR, cystic fibrosis transmembrane conductance regulator; LQ, limit of quantification; SIV, simian immunodeficiency virus; TSSM, tris, sodium, sucrose, mannitol.

Vector integration into the genomic DNA of transduced cells was detected in 6.7% (median) of NHP lung epithelial cells (Figure 2A). This was reproducible when comparing the three vector-treated NHPs individually (Figure 2B) and when comparing different regions of the lungs (Figure 2C). High levels of vector-specific messenger ribonucleic acid (mRNA) (median copy number: 1.1e6/μg total RNA) were detectable in NHP lung epithelial cells (Figure 3A). This was reproducible when comparing the three vector-treated NHPs individually (Figure 3B) and when comparing different regions of the lungs (Figure 3C). When ratioed to endogenous CFTR mRNA, vector-specific mRNA was ∼45-fold higher than endogenous CFTR levels **(**Figure 3D) and reproducible across individual NHPs (Figure 3E). Vector-specific mRNA was also detected in bronchial brushings (median copy number: 1.3e6/μg total RNA) (Figure 4A). These levels were ∼16-fold above endogenous CFTR mRNA levels (Figure 4B).

**Figure 5:**
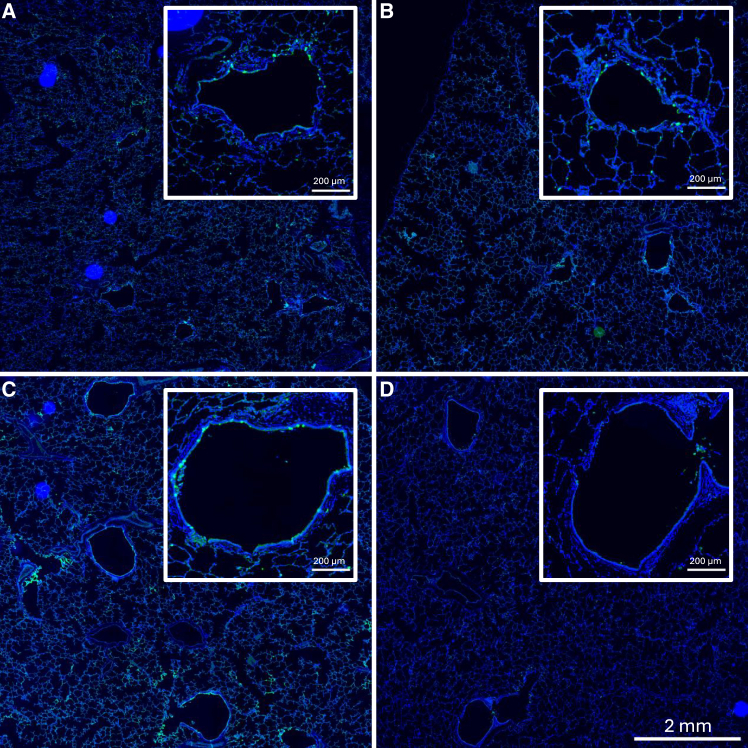
Protein expression in airway epithelial cells Representative immunofluorescence micrographs (A–D) of lung cross-sections containing multiple conducting airways, obtained from the three vector-treated animals (A, B, and C) and one diluent-treated animal (D) (with an immunofluorescence-stained lung section). Inset panels show magnified airways. Scale bars indicate corresponding real-world measurements. Green indicates EGFP; blue indicates DAPI-stained nuclei. DAPI, 4′,6-diamidino-2-phenylindole; EGFP, enhanced green fluorescent protein.

Enhanced green fluorescent protein (EGFP) immunohistochemistry was performed to quantify protein expression in airway epithelial cells (Figure 5). Transduced cells were detected in approximately 97% of airways analyzed (61 of 63 airways analyzed in three NHPs), and the mean airway epithelial cell transduction efficiency was 10.8% (8.7%, 12.2%, and 11.5% in individual NHPs) (Table 2) when quantified with ImageJ after airway segmentation.

**Table 2:** Mean percentage of airway epithelial cells expressing EGFP in vector-treated animals

Animal	Number of airways analyzed	Mean % of airway cells expressing EGFP
Animal 1	21	8.7
Animal 2	25	12.2
Animal 3	17	11.5
**All animals**	**63**	**10.8**

Vector-treated animals: *n* = 3. EGFP, enhanced green fluorescent protein.

## Discussion

Gene therapy is a mutation-agnostic approach that may benefit people with CF. We have developed a lentiviral vector pseudotyped with rSIV.F/HN as a potential treatment for CF. Here, we present the results of a study assessing the safety and efficacy of the vector in NHPs.

The data demonstrate tolerability and efficient transduction in respiratory tissues of NHPs following a single aerosolized dose of the rSIV.F/HN vector. Vector-related minimal mixed-cell centriacinar inflammation was observed in one vector-treated animal. However, this inflammation is a common finding seen upon exposure to test articles in inhalation studies^23^ and could also be incidental. This, in addition to the minimal nature of the finding, suggested this was a non-adverse observation.

The vector transduced ∼11% of airway epithelial cells, as assessed by EGFP immunohistochemistry. In this study, we did not use cell type-specific markers to identify which airway epithelial cells were transduced. However, we have previously shown that, in murine lungs, various airway epithelial cells as well as type 1 and 2 pneumocytes were transduced by the vector.^24^

Additionally, vector-specific mRNA levels were ∼45-fold higher than endogenous CFTR mRNA levels. Although uncommon, there are several risks posed by CFTR overexpression, such as abnormalities in cellular differentiation and proliferation (as per *in utero* studies),^25^ aberrant localization and cell polarization,^26^^,^^27^ and changes in ion transportation.^27^ However, the risk of CFTR overexpression with rSIV.F/HN is theorized to be low, and when extrapolating from results using an EGFP-expressing vector in this study, vector-specific CFTR mRNA expression with the use of rSIV.F/HN is predicted to provide clinically relevant correction of ion transport. Indeed, previous preclinical studies indicate that the restoration and normalization of CFTR-mediated chloride currents can occur if 5%–25% of airway epithelial cells are corrected,^24^ which compares favorably with the ∼11% of cells that were transduced in this experiment. In a practical context, this is confirmed by people with CF who have residual function mutations: in people with these mutations, those who retain 10% of normal CFTR expression (per cell) are typically unaffected by disease.^28^^,^^29^ The number of animals used in this study is comparatively small: for ethical reasons, studies in NHPs are generally conducted in small numbers of animals.

The 7-day tolerability data in NHPs are in line with those previously reported in mice.^24^ No gross tissue abnormalities were identified, and no changes in cytokine levels were observed. Additionally, several hematological parameters were changed between the groups, but these differences were small and inconsistent between time points and were likely due to normal variation between animals and not vector related.

In a related mouse study, mice were given a dose of 1E7 TU/mouse via nasal instillation and were monitored for 24 months. The mice displayed no evidence of chronic toxicity during the study period (mortality and weight were similar in vector-treated and untreated mice, and no differences in any of the key histological markers were reported).^22^ Farrow et al. also reported that there was no evidence of an inflammatory response in NHP lungs following intrapulmonary administration of a VSVG-HIV vector.^18^ Similarly, Guggino et al. reported that AAV-mediated gene transfer to NHP lungs was safe.^30^

We did not assess genotoxicity in this study. Assessment of genotoxicity in animal models using third-generation lentiviral vectors is not feasible, because animals would need to be housed for many years. In preparation for the ongoing clinical trial, the genotoxic potential of our lentiviral vector platform was assessed in industry-standard *in vitro* immortalization assays.

Integration site analysis in a large organ such as the lung is not informative. The integration site will vary cell by cell, and in contrast to bone marrow studies, clonal expansion is unlikely and, importantly, was not observed in the histological analysis. In addition, the amount of DNA analyzed in a reaction is miniscule compared with total DNA in the lung, and any insertion site analysis would only provide a very small snapshot. However, in preparation for the ongoing clinical trial, we have conducted insertion site analysis in human air-liquid interface cultures. This analysis did not indicate any hot spots or integrations near known proto-oncogenes.^29^

This non-GLP study was not designed to assess off-target expression using molecular assays. We have previously assessed non-target organ gene expression in mice dosed using intranasal administration of the vector and have not observed any evidence of protein expression other than, as expected, in nasal epithelium (U.G., personal communication).

This study was conducted with an rSIV.F/HN vector carrying a GFP reporter rather than the CFTR cDNA, because the specificity of CFTR antibodies is generally poor. In addition, we are not aware of any CFTR antibodies that discriminate human from NHP CFTR. For these reasons, the use of a lentiviral vector expressing human or mouse CFTR was not feasible. Analysis was performed 7 days after transduction, because NHPs develop immune responses to non-self transgenes, which impact on the duration of expression.^31^^,^^32^

When carrying the *CFTR* transgene, the rSIV.F/HN vector has previously demonstrated expression of functional CFTR activity *in vitro*^3^^,^^24^ and partial restoration of CFTR function in CF organoids.^24^ Indeed, the vector transduced fully differentiated airway epithelium, producing functional CFTR chloride channels^3^; furthermore, rSIV.F/HN-hCEF-CFTR-treated intestinal organoid cultures showed evidence of significant increases in organoid swelling when compared with negative controls.^24^

Although CFTR modulator therapy has produced exceptional improvements in clinical outcomes and quality of life in people with CF,^33^^,^^34^ a considerable proportion (10%–15%) of people with CF are ineligible for, or intolerant to, CFTR modulator therapy,^1^^,^^35^ underscoring the importance of a mutation-agnostic treatment for CF. To address this treatment need, several gene therapies using both non-viral and viral technology have been investigated in clinical trials for the treatment of CF; however, none of these therapies have reached the market.^35^^,^^36^ It should be noted that the dose examined in this study does not allow for conclusions on human-related doses; however, the importance of NHPs as a translational model, with their high degree of genetic and physiological similarity to humans,^37^ remains valid in the context of this research.

The rSIV.F/HN vector carrying a codon-optimized and CpG-depleted CFTR cDNA (BI 3720931) is being assessed in the first-in-human clinical trials (Lenticlair 1 [NCT06515002]^38^ and Lenticlair-ON [NCT06962852]^39^) in people with CF who are ineligible for CFTR modulator therapy. Lenticlair 1 is a phase 1/2 trial wherein people with CF will receive a single inhaled dose of BI 3720931 alongside standard-of-care therapy. BI 3720931 is being delivered via oral inhalation. The primary objective is to investigate safety and tolerability of a single inhaled dose of BI 3720931 based on the number of trial participants who experience at least one drug-related, treatment-emergent adverse event (AE) up to 24 weeks after dosing. The primary endpoint for the trial is the occurrence of any drug-related treatment-emergent AEs within the study period, while secondary endpoints will examine the effect of BI 3720931 on multiple clinical endpoints, including those relating to lung function. Lenticlair-ON is an extension trial that will then monitor long-term safety in treated participants. In this study of NHPs, vector-specific mRNA was detected not only in the lung tissue of NHPs but also in bronchial brushings. This is of relevance, as gene expression is being monitored in bronchial brushings and biopsies collected during the phase 1 part of Lenticlair 1. Should BI 3720931 yield favorable outcomes, the unmet treatment need in non-modulator-treated people with CF may finally be addressed.

### Conclusions

rSIV.F/HN demonstrated transduction efficiency in NHPs, with no evidence of relevant toxicity with use of the EGFP-based vector. These NHP-related data, together with previous murine data, support further progression of BI 3720931 toward the clinic, with a first-in-human trial of BI 3720931 (Lenticlair 1) having begun in late 2024.

## Materials and methods

Animal studies were conducted at Lovelace Biomedical, Albuquerque, New Mexico, USA. The Institutional Animal Care and Use Committee at LoveLace Biomedical and the Animal Welfare Ethical Review Board at Imperial College and Edinburgh University reviewed and approved the study in line with US and UK guidelines.

Additionally, this study complied with all applicable sections of the Final Rules of the Animal Welfare Act regulations^40^ (Title 9 Code of Federal Regulations Parts 1, 2, and 3),^41^ as well as the Guide for the Care and Use of Laboratory Animals (2011).^42^ Lovelace Biomedical is fully accredited by the Association for Assessment and Accreditation of Laboratory Animal Care.

### Vector manufacturing

The lentiviral vector used in this study was developed by the Gene Medicine Research Group, University of Oxford, UK. Recombinant SIV vector was produced in HEK293T cells grown in suspension as previously described,^24^ following five-plasmid transient transfection with PEIpro (PolyPlus), including the vector genome expressing EGFP under the control of the hCEF promoter (a transgene promoter composed of cytosine guanine dinucleotide (CpG)-free CMV enhancer/elongation factor 1 alpha promoter).^24^ Vectors were purified using anion exchange chromatography and tangential flow filtration and formulated into TSSM buffer. The functional titer in TU/mL was determined based on quantifying the genomic integration of woodchuck hepatitis virus post-transcriptional regulatory element (WPRE) DNA sequence after transduction^43^ in the absence of polybrene.

### Aerosol administration

The first animal was anesthetized with an intramuscular injection of Telazol (5–8 mg/kg; 100 mg/mL; Dechra, Leawood, KS, USA), which led to deep anesthesia, but required a long recovery time. Subsequent animals were anesthetized with ketamine (5–10 mg/kg; 100 mg/mL; VetOne, Boise, ID, USA) via intramuscular injection because the time needed to aerosolize the vector was comparatively short (minutes).

A cuffed endotracheal tube (VetOne, Boise, ID, USA) was placed in the trachea, directly below the larynx, and secured to avoid slipping out. Thereafter, animals received intramuscular injections of ketamine (5–10 mg/kg), if needed, to keep them anesthetized for the duration of the inhalation procedure.

Animals were intubated, and an Aerogen Solo nebulizer (Aerogen, Chicago, IL, USA; for aerosol generation), in conjunction with an AeroEclipse II nebulizer (Trudell Medical International, London, ON, Canada; for controlled inhalation delivery) and an Aerogen Ultra (Aerogen, Chicago, IL, USA) device (holding chamber), was used to aerosolize and deliver the viral vector formulation to NHPs through an endotracheal tube.

### Aerosol method development

Prior to initiation of the *in vivo* studies, the following aerosol method development studies were conducted.

(1) The delivery efficiency of the nebulizer system was calculated using the following formula (Equation 1):
  (Equation 1) $$Delivery\;efficiency\;\left(\%\right)=\frac{Amount\;of\;viral\;formulation\;delivered\;to\;the\;end\;of\;the\;ET\;tube}{Amount\;of\;test\;article\;added\;to\;the\;nebuliser}\times 100\text{.}$$
(2) Tests were performed to determine the effect of aerosolization on the infectivity of the viral vector. Following aerosolization and condensation, transduction efficiency of condensate was analyzed in A549 cells *in vitro*, and the infectivity of the stock viral agent was compared with that of the condensate.

### Determination of vector delivery and deposition

The delivered amount of vector was calculated using the following formula, where only a fraction of the delivered product was expected to deposit in the NHP respiratory tract (Equation 2):

(Equation 2) Deliveredamountofvector(TU/animal)=amountoftestarticleused×deliveryefficiencyofsystem.

The deposited amount of the viral vector was then estimated using the following equation, based on a deposition fraction of 0.25 for NHPs (as per the US Food and Drug Administration’s Division of Pulmonary, Allergy and Rheumatology Products-accepted deposition fractions) (Equation 3)^44^:

(Equation 3) Despositionofvector(TU/animal)=deliveredamountofvector×depositionfraction.

### Experimental design

Male cynomolgus monkeys received a single aerosolized dose of rSIV.F/HN expressing EGFP (*n* = 3; 4.2E9 TU in 2.1 mL Tris [20 mM; pH 7.3], sodium [100 mM], sucrose [10 mg/mL] and mannitol [10 mg/mL] [TSSM] diluent^43^) or diluent only (*n* = 3; 2.1 mL TSSM). The time taken to deliver the vector or diluent was recorded. Seven days post-transduction, animals were first sedated through intramuscular injection of ketamine (10–20 mg/kg). After sedation, animals were euthanized with an overdose of a barbiturate-based sedative (≥1 mL/4.5 kg) injected intravenously.

### Observations and measurements

**Figure 6:**
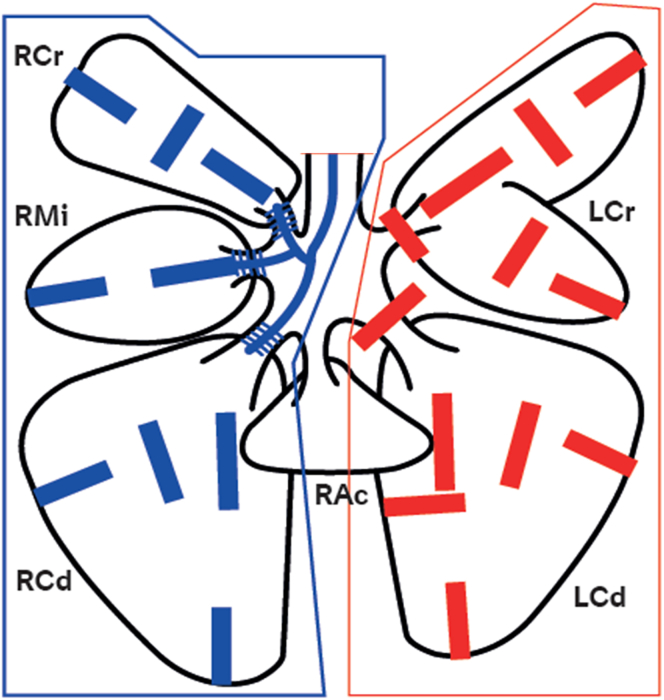
Approximate tissue sampling sites in NHP lungs Blue, sites for molecular analyses; red, sites for histopathology and immunohistochemistry. The blue brushes indicate the regions where bronchial brushings were taken from. LCd, left caudal; LCr, left cranial; NHP, non-human primate; RAc, right accessory; RCd, right caudal; RCr, right cranial; RMi, right middle.

Body weight was recorded and blood samples collected prior to exposure, and animals were monitored for clinical signs post-exposure. Animals were euthanized 7 days post-dosing. At necropsy, blood samples were collected and body and organ weights (brain, heart, kidney, liver, lung, and spleen) were recorded. Extensive lung sampling was performed from the areas shown in Figure 6. These measurements and samples were used for the following analyses: histopathology, clinical pathology, cytokine quantification, and changes in body and organ weight. Quantitative polymerase chain reaction (qPCR) to determine vector genome integration into host cell DNA, quantitative reverse-transcription PCR (RT-qPCR) to quantify vector-specific mRNA and endogenous CFTR mRNA, and EGFP immunohistochemistry for transgene expression were performed on lung tissues.

Serum samples collected for cytokine analyses were analyzed using customized U-PLEX assay kits (Mesoscale Discovery, Meso Scale Diagnostics LLC, Rockville, MD, USA). The following cytokines were evaluated: granulocyte colony-stimulating factor, granulocyte-macrophage colony-stimulating factor, interferon (IFN)-α2a, IFN-γ, interleukin (IL)-1β, IL-1RA, IL-2, IL-4, IL-5, IL-6, IL-8, IL-10, IL-13, IL-15, IL-17A, IL-18, IL-12/IL-23p40, IFN-inducible T cell alpha chemoattractant, monocyte chemoattractant protein-1, macrophage inflammatory protein-1 alpha, macrophage inflammatory protein-1 beta, tumor necrosis factor alpha, and vascular endothelial growth factor A.

For the immunohistochemistry analysis, formalin-fixed paraffin-embedded lung tissue sections were prepared, immunofluorescence using anti-GFP antibodies was performed, and microscope images were captured. Using Fiji (ImageJ) software, a semi-automatic macro was applied to segment contiguous regions of airway epithelium and to calculate the percentage of EGFP-positive and -negative regions. Conducting airway epithelial border analysis segmentations of a fixed pixel thickness was used to encompass an approximate single-cell layer. It was confirmed through visual qualitative analysis that this approximation could be used to report EGFP percentage area as a proxy for percentage of EGFP-positive airway epithelial cells.

All DNA for qPCR was isolated using a MagMAX-96 DNA Multi-Sample Ultra 2.0 Kit (Cat# A36570; Thermo Fisher Scientific, Waltham, MA, USA), and all RNA for RT-qPCR was isolated using a Direct-Zol 96-RNA Kit (Cat# R2102; Zymo Research, Irvine, CA, USA). The WPRE primer sequences used to amplify integrated vector DNA and vector-specific mRNA were Forward: 5′ TGG-CGT-GGT-GTG-CAC-TGT 3′ and Reverse: 5′ CCC-GGA-AAG-GAG-CTG-ACA 3′, and the conditions for amplification on the Bio-Rad CFX instrument were 40 cycles of denaturation at 95°C for 3 s and annealing/extension at 60°C for 30 s. The commercial NHP CFTR assay kit from Applied Biosystems (Thermo Fisher Scientific, Waltham, MA, USA; assay ID: Mf02787638_m1; Cat# 4448489) was used to assess expression from the endogenous NHP *CFTR* gene. The padded amplicon sequence (FASTA format) of the NHP *CFTR* gene (*Macaca fascicularis* CFTR, transcript variant X1, mRNA) was as follows:

>XM_005550598.2:140–440 predicted: GTCGCCTCTGGAAAAGGCCAGCGTTGTCTCCAAACTTTTTTTCAGCTGGACCAGACCAATTTTGAGGAAAGGATACAGACAGCGCCTGGAATTGTCAGATATATACCAAATCCCTTCTGCTGATTCTGCTGACAATCTATCTGAAAAATTGGAAAGAGAATGGGATAGAGAGCTGGCTTCAAAGAAAAATCCCAAACTCATTAATGCCCTTCGGCGATGCTTTTTCTGGAGATTTATGTTCTATGGAATCTTGTTATATTTAGGGGAAGTCACCAAAGCAGTACAGCCTCTCTTACTGGGA.

Conditions for this procedure were the same as described above.

### Statistical methods

Parametric or non-parametric distribution of data was confirmed. Statistical tests suitable for analysis of two-group or multiple-group parametric data (*t* test and analysis of variance) or two-group or multiple-group non-parametric data were performed as appropriate. The null hypothesis was rejected at *p* < 0.05.

## Data and code availability

The datasets used and/or analyzed during the current study are available from the corresponding author on reasonable request.

## Acknowledgments

The authors would like to acknowledge Adriana Rascon for contributions to the PCR sample analysis and data interpretation. J.C.D., U.G., and E.W.F.W.A. would like to acknowledge the 10.13039/501100000272National Institute for Health and Care Research through the 10.13039/501100013342Imperial Biomedical Research Centre. J.C.D. and E.W.F.W.A. are NIHR senior investigators.

## Author contributions

All authors contributed to the writing of the manuscript. E.C., R.J.D., M.A.V., D.R.G., and S.C.H. designed the vector system and manufactured the test article. In addition, A.G. performed the histopathology and data analysis; K.V. performed histopathology analyses; A.S. supported with immunohistochemistry analyses; C.C., J.A., and D.B. supported with strategy, study design, and data interpretation; D.S. contributed to the management, planning, and execution of the in-life phase and supported with data reporting and interpretation; U.G., G.M., A.S., K.P., A.C.B., and E.W.F.W.A. supported the design and oversight of the study and conducted data analysis; J.C.D., M.C., and C.M. contributed to the execution of aerosol exposures and supported with aerosol data reporting and interpretation.

## Declaration of interests

U.G., G.M., A.C.B., and J.C.D. declare patents and royalties or licences related to the submitted work and consulting fees and support for attending meetings and/or travel from Boehringer Ingelheim. D.R.G. and S.C.H. declare patents and royalties or licences related to the submitted work and consulting fees from Boehringer Ingelheim. C.C., J.A., and A.G. are employees of Boehringer Ingelheim. D.B. was an employee of Boehringer Ingelheim at the time of this study. E.W.F.W.A. declares patents and royalties or licences related to the submitted work, and consulting fees, support for attending meetings and/or travel, and participation on a data safety monitoring board or advisory board for Boehringer Ingelheim.

This study was part-funded by Boehringer Ingelheim International GmbH. The authors did not receive payment related to the development of the manuscript. Laura Cottino, PhD, of Nucleus Global, provided writing, editorial support, and formatting assistance, which was contracted and funded by Boehringer Ingelheim. Boehringer Ingelheim was given the opportunity to review the manuscript for medical and scientific accuracy as well as intellectual property.
